# Grain and Leaf Anthocyanin Concentration Varies among Purple Rice Varieties and Growing Condition in Aerated and Flooded Soil

**DOI:** 10.3390/molecules27238355

**Published:** 2022-11-30

**Authors:** Pennapa Jaksomsak, Sawika Konseang, Bernard Dell, Hatem Rouached, Chanakan Prom-u-thai

**Affiliations:** 1 Program in Agricultural Chemistry, Faculty of Agricultural Production, Maejo University, Chiang Mai 50290, Thailand; 2 Agricultural and Forestry Sciences, Murdoch University, Murdoch, WA 6150, Australia; 3 Plant Resilience Institute, Michigan State University, East Lansing, MI 48824, USA; 4 Department of Plant, Soil and Microbial Sciences, Michigan State University, East Lansing, MI 48824, USA; 5 Agronomy Division, Department of Plant and Soil Sciences, Faculty of Agricultural, Chiang Mai University, Chiang Mai 50200, Thailand; 6 Lanna Rice Research Center, Chiang Mai University, Chiang Mai 50200, Thailand

**Keywords:** purple rice grain, water management, health benefit, pigmented rice

## Abstract

Anthocyanins are a group of pigments responsible for the red-blue color in plant parts, and have potential for health benefits and pharmaceutical ingredients. To evaluate whether anthocyanin concentrations in five purple rice varieties could be varied by water condition, plants were grown in waterlogged and aerobic (well-drained) soil. Grain anthocyanin concentration and grain yield were measured at maturity, while leaf anthocyanin concentrations were measured at booting and flowering stages. Four varieties grown under the waterlogged condition had 2.0–5.5 times higher grain anthocyanin than in the aerobic condition. There was a positive relationship between grain and leaf anthocyanin at booting in the waterlogged condition (r = 0.90, *p* < 0.05), while grain and leaf anthocyanin were positively correlated at flowering in both the waterlogged (r = 0.88, *p* < 0.05) and aerobic (r = 0.97, *p* < 0.01) conditions. The results suggest that water management should be adopted as a practical agronomic tool for improving the anthocyanin concentration of purple rice for specialist markets, but the specific responses between rice varieties to water management should be carefully considered.

## 1. Introduction

In purple rice, anthocyanin pigments accumulate in the grain pericarp and their expression in other plant parts varies with rice varieties. Some varieties have anthocyanins in leaves, leaf sheath, stigma and pericarp, and others in all above-ground plant parts [1,2]. Recently, anthocyanins have gained commercial attention due to potential beneficial health effects in food and pharmaceuticals [3,4,5,6]. The major health benefits of Thai purple rice relate to their free radical scavenging, anti-hyperlipidemic, anti-cancer and anti-inflammatory properties [7,8,9]. Furthermore, it is claimed that anthocyanins in rice and other plants also are effective cosmetic ingredients for preventing skin aging, UV induced skin damage and hair loss [10,11,12]. Thus, cultivation of rice varieties with high anthocyanin concentration could provide an opportunity for producers to enhance the profitability of their farms.

There is wide variation in grain anthocyanin concentration among the purple rice germplasm in Thailand. For example, the anthocyanin concentration ranged from 9.8–245.4 mg 100 g^−1^ in purple rice varieties collected from southern Thailand [13]. Local purple rice germplasm from the highlands in northern Thailand have high anthocyanin (22.7 ± 77.3 mg 100 g^−1^) as well as possess other nutritional qualities such as high Fe and Zn contents and anti-oxidative capacity [14]. Furthermore, several studies have established that purple rice varieties have high phenolic acid and flavonoid contents, and high antioxidant capacity compared with red and white pericarp rice varieties [15,16,17]. Therefore, it is claimed that purple rice varieties are a quality source of nutrients and are rich in bioactive compounds that are beneficial to human health [18]. Apart from the observed genetic variation in pigmentation among rice varieties, environmental factors can affect the anthocyanin concentration in the other plant organs. Thus, understanding factors controlling anthocyanin accumulation in rice would assist producers to optimize the production of high anthocyanin rice for their health benefits.

Anthocyanins play a major role in plant protection under adverse environments such as ultraviolet radiation, temperature, nutrient imbalances and low or excess water conditions [19,20,21]. Growing rice plants at different altitudes, as in lowland and highland locations, has been reported to influence grain anthocyanin concentrations among purple rice varieties and this was attributed to differences in soil nutrients and soil moisture or the micro-climate between the different altitudes [22]. Furthermore, anthocyanin concentrations in rice plant parts may be affected by agronomic practices as high N fertilizer rates promoted anthocyanin accumulation in the shoot but not in the grain [23]. Water is essential for physiological processes in plants, including growth, ontogeny and metabolism. However, its supply is not always optimal and the role of water in anthocyanin biosynthesis has not been investigated. Whether water management affects anthocyanin accumulation in purple rice is unknown. Given that purple rice varieties can be successfully grown under rain-fed and irrigated conditions, it is useful to investigate how waterlogged and aerobic conditions influence grain and leaf anthocyanin concentration among purple rice varieties. The hypothesis was addressed that anthocyanin in grain and leaves in different purple rice varieties could be stimulated by suitable water management. The research findings in this study will be of benefit to rice breeders and producers.

## 2. Results

Grain yield and grain anthocyanin concentration in brown rice were significantly affected by variety and water and there was an interaction effect between variety and water regimes (Table 1).

**Figure 1:**
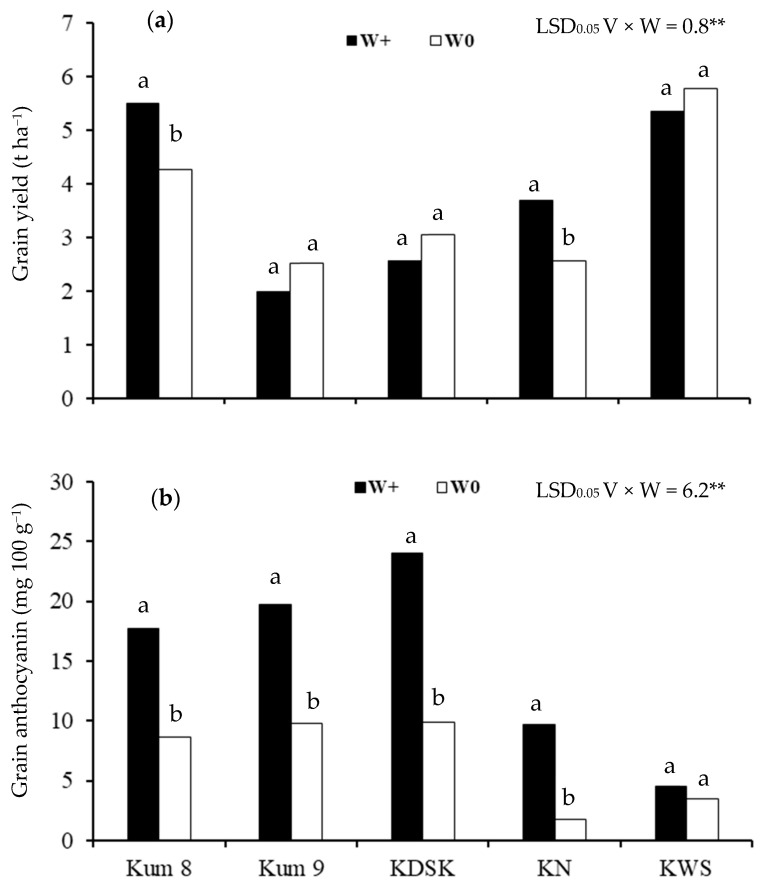
Grain yield (**a**) and grain anthocyanin concentration (**b**) of 5 purple rice varieties grown under 2 water regimes (waterlogged, W+; aerobic, W0) (Significant interaction effect of variety × water regime at *p* < 0.01 **). Different lowercase letters above the bars indicate least significant differences between water condition in each variety at *p* < 0.05.

Among the five varieties, grain yield ranged from 2.0–5.5 t ha^−1^ in the waterlogged condition and 2.5–5.8 t ha^−1^ in the aerobic condition (Figure 1a). Grain yields of Kum 8 and KN were 1.3 and 1.4 times higher, respectively, in the waterlogged than in the aerobic soil. There was no effect of water regime on grain yield in the other three varieties.

Grain anthocyanin concentration ranged from 4.6 to 24.0 mg 100 g^−1^ in plants grown in the waterlogged condition and from 1.8 to 9.8 mg 100 g^−1^ in the aerobic condition (Figure 1b). Concentrations in Kum 8, Kum 9, KDSK and Kum Na were 2.1, 2.0, 2.4 and 5.5 times higher, respectively, in waterlogged plants than in plants grown in the aerobic condition, but there was no effect of water regime in KSW.

**Figure 2:**
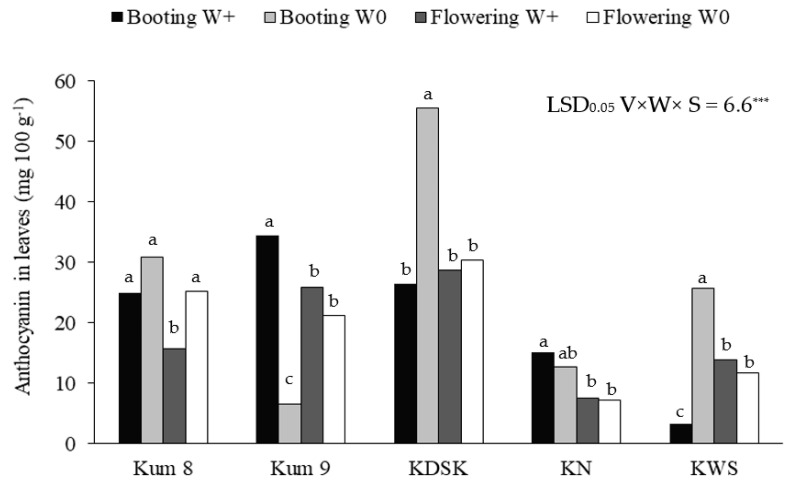
Anthocyanin concentrations in leaves at booting and flowering stages of 5 purple rice varieties grown under waterlogged (W+) and aerobic (W0) conditions (Significant interaction effect of variety × water regime × growth). Significant interaction effect of variety × water regime × growth stage at *p* < 0.001 ***. Different lowercase letters above the bars indicate least significant differences between treatment means in each variety at *p* < 0.05.

**Figure 3:**
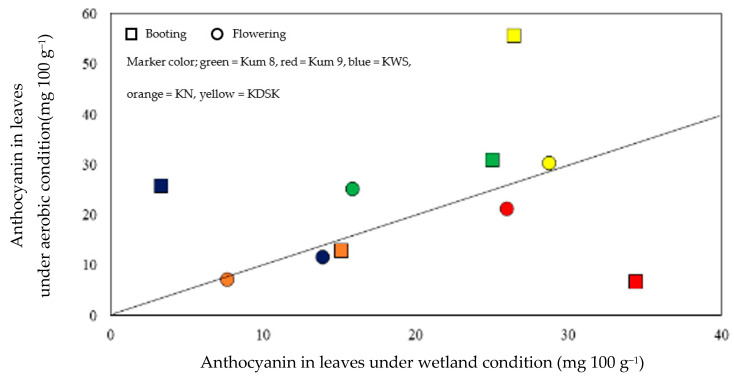
Comparison of anthocyanin in leaves at booting and flowering stages of 5 purple rice varieties grown under waterlogged compared with aerobic condition. Each data point is the mean of 3 replications.

Leaf anthocyanin concentrations were affected by variety, water and growth stage, and interaction effects between the three factors were also observed (Table 1, Figure 2). The leaf anthocyanin concentrations ranged from 3.3 to 34.4 mg 100 g^−1^ in the waterlogged condition and from 6.6–55.6 mg 100 g^−1^ in the aerobic condition. At booting, leaf anthocyanin ranged from 3.3–55.6 mg 100 g^−1^, and at flowering the range was 7.3–30.3 mg 100 g^−1^ (Figure 2). Under the aerobic condition, leaf anthocyanin concentrations at booting in KDSK and KWS were 2.1 and 7.8 times higher, respectively, than in waterlogged plants. Moreover, at flowering, the leaf anthocyanin concentration in Kum 8 was 1.6 times higher in aerobically grown plants (Figure 3). In contrast, leaf anthocyanin concentration in Kum 9 at booting was depressed by 5.2 times in the aerobic condition compared with plants grown in the wetland condition. However, water regime had no effect on leaf anthocyanin concentration in KN. However, the water regime had no effect on leaf anthocyanin concentration in KN.

**Figure 4:**
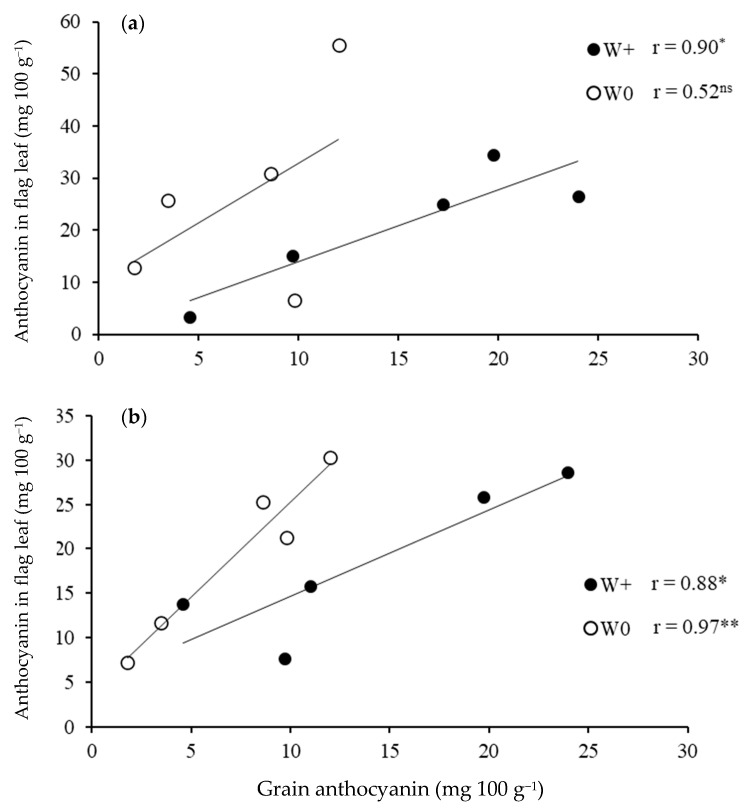
Correlation between grain anthocyanin and anthocyanin in leaves at booting (**a**) and flowering (**b**) under waterlogged (W+) and aerobic conditions (W0). Each data point is the mean of 3 replications. (Significant correlation at *p* < 0.05 * and *p* < 0.01 **).

Grain yield was not associated with the concentrations of anthocyanin in the grain or in leaves at booting and flowering stages in either water regimes (Data not shown). On the other hand, there was a significant positive correlation between grain and leaf anthocyanin concentrations at booting in the plants grown in the waterlogged condition (r = 0.90 at *p* < 0.05), but there was no correlation in the aerobic condition (Figure 4a). At flowering, grain anthocyanin was positively correlated with leaf anthocyanin in both the waterlogged (r = 0.88, *p* < 0.05) and aerobic (r = 0.97, *p* < 0.01) conditions (Figure 4b).

## 3. Discussion

This experiment showed that grain and leaf anthocyanin concentrations were influenced by water regimes, but the responses were affected by rice variety and growth stage for the leaf anthocyanin concentration. Grain anthocyanin in four of the five purple rice varieties increased substantially when grown in the waterlogged condition compared with the aerobic condition. The 2.0–5.5 times increase occurred without a dilution effect from grain yield as indicated by there being no correlation between grain anthocyanin and yield. Unlike for the grain of plants grown in the wetland soil, both positive and negative responses in leaf anthocyanin were found among rice varieties and growth stages. Furthermore, there were positive relationships between grain and leaf anthocyanin concentration in both the waterlogged and aerobic plants at flowering but only in waterlogged plants at booting.

Anthocyanin metabolism in plants is regulated by genes which could be induced by environmental stresses such as ultraviolet radiation, temperature extremes and water deficit at specific development stages in vegetative and reproductive organs [24,25,26]. Responses of anthocyanin biosynthesis to unfavorable water conditions have been reported in cereal crops. In purple wheat, mild drought stress altered grain anthocyanin without an impact on grain yield, whereas severe drought stress increased both grain yield and anthocyanin at the late grain filling stage [27]. Furthermore, it has been reported that drought slightly decreased grain anthocyanin with upregulation and downregulation of anthocyanin biosynthesis related genes occurring at the same time in purple wheat [21]. A range of environmental factors have been shown to alter the accumulation of anthocyanins in grain and leaves of purple rice. For example, foliar application of MgSO4 increased grain and leaf sheath anthocyanin in Hom nil, an improved purple rice variety in Thailand, which were grown under 16 °C air temperature for 28 days [28]. In purplish leaf varieties, salt stress enhanced anthocyanin levels in rice seedlings and simultaneously increased the level of gene expression involved in anthocyanin biosynthesis [29]. Decreasing the water supply in containers, by alternate wetting and drying irrigation (AWD), markedly increased the anthocyanin content in the riceberry variety of purple rice [30]. By contrast, our results found that growing plants in aerobic soil reduced the grain anthocyanin in most of the purple rice varieties originating as wetland ecotypes. Factors that may have contributed to this response include the water status of the plants not reaching a threshold to trigger the protective mechanism of anthocyanin pigmentation in grain, and genotypic variation of this mechanism among purple rice varieties. Further research is needed to explore the physiological, biochemical and molecular responses of purple rice under different water regimes to better understand grain anthocyanin accumulation.

Leaf anthocyanin concentrations were more variable than in the grain. Anthocyanin biosynthesis in vegetative and reproductive parts are governed by different gene suites [31]. For example, anthocyanin biosynthesis in most organs of rice, except the pericarp, was activated by the R2R3-MYB transcription factor (TF), OsC1 [32]. Furthermore, OsC1, OsRb and OsDFR were identified as the determinants of anthocyanin biosynthesis in rice leaves [33]. It has been reported that the genes Rd, OsCH and Kala4 are involved in anthocyanin biosynthesis in the rice pericarp [34]. The influence of water regimes on gene expression, anthocyanin synthesis and storage in rice remain to be determined.

Grain anthocyanin concentration was affected by an interaction between variety and water condition in this study. The grain anthocyanin concentration was highly correlated with the anthocyanin concentration in the leaf, especially at the flowering stage. Appropriate water management could help to increase synthesis and accumulation of anthocyanin in the leaf and subsequently improve the grain anthocyanin content in purple rice varieties. A similar effect of variety × environment on proanthocyanidin contents and total antioxidant capacity was found in red rice varieties [35]. The results from this study provide useful information on the selection of purple rice varieties and cultivation method to obtain high-quality grain for the end users.

## 4. Materials and Methods

### 4.1. Rice Varieties and Cultivation

**Figure 5:**
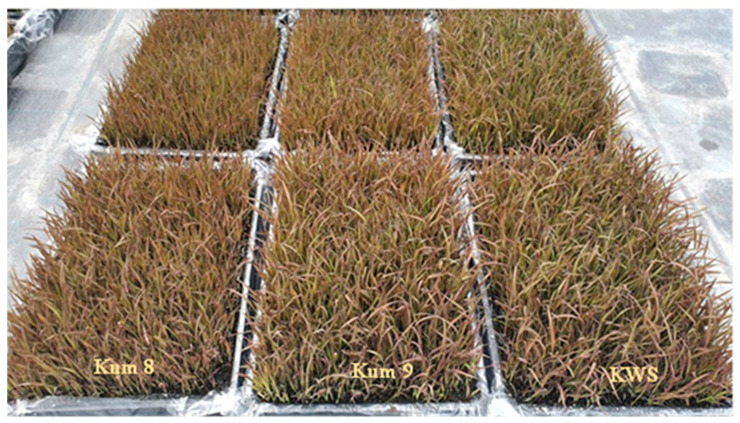
Purple rice seedling of Kum 8, Kum 9 and KWS used in this experiment.

Five purple rice varieties (Kum 8, Kum 9, KDSK, KN and KWS provided by the Agronomy Division, Chiang Mai University, Thailand), photoperiod sensitive and wetland ecotypes, and with anthocyanin pigments in all shoot parts, were used for this research (Figure 5). The varieties were local purple rice commonly grown in northern Thailand. Field experiments were conducted during the wet season (July to December) at Chiang Mai University, Thailand. Soil physical and chemical characters were as follows: soil texture; sandy loam, pH (soil: water, 1:1) 6.3, organic matter 1.6%, available P 51.0 mg kg^−1^ (Bray II), exchangeable K 120.3 mg kg^−1^ (NH_4_OAc) and extractable Zn 1.2 mg kg^−1^. They were grown in two water conditions: waterlogged cultivation (water level kept at 10–15 cm above the soil surface until 14 days before harvesting) and aerobic cultivation (irrigated daily and allowed to drain). One-month-old seedlings of each variety were transplanted in 2 × 2 m plots at 0.25 × 0.25 m spacing between hills with three replications. Fertilizers were applied to all plots on a surface area basis at the rate of 30 kg N ha^−1^ (as urea), 30 kg P_2_O_5_ ha^−1^ (as triple super phosphate) and 30 kg K_2_O ha^−1^ (as potassium chloride) at 15 days after transplanting, followed by 20 kg N ha^−1^ at panicle initiation. Youngest emerged leaf blade samples were collected at booting and flowering stages, placed on ice, stored at −20 °C and then freeze dried. Panicles were taken at maturity and grain yield was measured at 14% moisture content. The unhusked grain samples were de-husked by a laboratory husker (Model P-1 from Ngek Seng Huat Co., Ltd., Bangkok, Thailand) to produce brown rice (caryopsis) and then freeze dried.

### 4.2. Anthocyanin Analysis

Sub samples of freeze-dried leaves were mechanically ground in a hammer mill and monomeric anthocyanin measured by the pH-differential method [36]. About 1.0 g of leaf and 2.5 g of whole grain of brown rice samples were extracted in double deionized water (DDI) at 50 °C for 30 min. The extracted solution was filtered through Whatman grade 1 paper, transferred to volumetric flasks and then adjusted with potassium chloride buffer (pH 1.0) and sodium acetate buffer (pH 4.5). Each dilution was allowed to equilibrate for 15 min. The absorbance of anthocyanin pigment in 2 dilutions was measured at 520 and 700 nm using a spectrophotometer (Biochrom Libra S22, London, UK), against a blank cell filled with distilled water. The anthocyanin pigment concentration in the sample was determined with the following formula:

Anthocyanin pigment (cyanidin-3-glucoside) = (A × MW× DF × 1000)/(ε × L)

where A = (A520–A700) pH 1.0 − (A520–A700) pH 4.5; MW, molecular weight of cyanidin-3-glucoside = 449.2 (g/mol), DF = dilution factor; ε, molar absorptivity of the pigment = 26,900 molar extinction coefficient, in L × mol ^−1^ × cm^−1^ and L = 1 cm for cell path length.

### 4.3. Statistical Analysis

Analysis of variance was conducted to determine the effect of each factor and interaction between factors for grain yield, grain and leaf anthocyanin concentration using Statistix 8 (analytical software, version SXW, Tallahassee, FL, USA). The least significant difference (LSD) at *p* < 0.05 was applied to compare the means for significant differences. The relationship between data sets were analyzed with linear correlation.

## 5. Conclusions

Grain and leaf anthocyanin accumulation of purple rice varieties varied with the water regime. In general, grain anthocyanin concentrations were higher in plants grown in waterlogged than in aerobic soil but there were differences between varieties. Therefore, water management should be carefully considered during cultivation of purple rice varieties as high anthocyanin concentrations may increase the return to growers. This is of particular interest to producers interested in growing functional foods with high nutritional values for niche markets. Further research is required to determine the mechanisms by which the water regime affects the biosynthesis and storage of anthocyanin in the rice plant.

## Figures and Tables

**Table 1:** Significant effects by ANOVA of variety, water and growth stage on grain yield and anthocyanin in grain and leaf of 5 purple rice varieties grown in waterlogged and aerobic condition.

Effect	Grain Yield	Anthocyanin in Grain	Anthocyanin in Leaf
Variety (V)	*p* < 0.001	*p* < 0.001	*p* < 0.001
Water (W)	*p* < 0.05	*p* < 0.05	*p* < 0.01
Stage (S)	-	-	*p* < 0.001
V × W	*p* < 0.01	*p* < 0.01	*p* < 0.001
V × S	-	-	*p* < 0.001
W × S	-	-	*p* < 0.05
V × W × S	-	-	*p* < 0.001

## Data Availability

Not applicable.

## Sample Availability

Samples of the compounds are available from the authors.
